# Who Will Take Care of Me?: Older Parents’ Practice and Plans for Their Future

**DOI:** 10.1093/geroni/igaa057.2985

**Published:** 2020-12-16

**Authors:** Noriko Toyokawa

**Affiliations:** California State University San Marcos, San Marcos, California, United States

## Abstract

The current study investigated older parents’ practices and plans to support their own autonomous lives with/without children’s living assistance and how these parents negotiate autonomous lives with their adult children among three racial/ethnic groups. The study participants were older parents who resided in the Southern California who were recruited through local senior centers (N = 15, Mage = 75, SD = 8.7; Caucasians 47%, Mexicans 40%, Filipinos 13%). Content analysis revealed that Caucasian American parents supported their autonomous lives by using friend networks and professional caregivers rather than depending on their children. Parents with Mexican origin emphasized that their children well took care of them and there were no conflicts with their children. Filipino parents planned to migrate to the Philippines where professional services were affordable so that they would not rely on their children’s care. Similarities and differences in older parents’ need among different racial/ethnic groups will be discussed.

